# Prenatal screening for Mendelian disorders in antenatal care

**DOI:** 10.1186/1755-8166-7-S1-I16

**Published:** 2014-01-21

**Authors:** Amar Verma

**Affiliations:** 1 Department of Paediatrics & Neonatology, Rajendra Institute of Medical Sciences, Ranchi, Jharkhand, India

## Antenatal screening for fetal abnormality should be offered to all women, if available

In all cases of antenatal screening, the woman must be fully informed and understand the implications of the test, be promptly advised of their test result and be referred for further management and definitive diagnosis if their screening test is positive or suggestive of high risk.

A positive prenatal diagnosis poses many ethical issues and challenging decisions for parents and clinicians. In those at increased risk of having a baby with a genetic condition, the risk should be identified and discussed fully before pregnancy and options for prenatal diagnosis discussed. Genetic counseling should be provided.

At the present time around 5000 known disorders are inherited in a monogenetic mendelian fashion. Foremost among them are autosomal dominant, autosomal recessive and X-linked disorders, which carry a higher risk of illness than that conveyed by age-related risk. An autosomal dominant condition carries an a priori 50% inheritance risk where one parent is affected. An autosomal recessive disease carries a 25% inheritance risk for children of a healthy carrier couple. An X-linked recessive disorder carries a 50% risk for the son of a carrier mother.

Specific, albeit non-screening genetic tests are currently available for more than 1000 of these diseases. Unlike cytogenetic, prenatal diagnosis based on maternal age, prenatal gene testing is not a screening test. Given the individuality of each case, prior planning is essential. Two differing strategies are possible: indirect and direct genetic testing.

The following subsections cover the antenatal screening tests that are routinely offered like screening for potential for neonatal infection, Haemolytic disease of new born, Sickle cell disease and Thalassaemia, Down's syndrome, Fetal anomaly, Measurement of fundal height etc.

The different types of tests like Biochemical, Cytogenetic and Molecular genetic tests can be carried out by Chorionic villus sampling, Amniocentesis, Cordocentesis / percutaneous umbilical blood sampling Fetoscopy, Fetal radiology, Ultrasound-guided percutaneous skin and organ biopsy, Maternal blood tests, Ultrasound-guided percutaneous skin and organ biopsy and Preimplantation prenatal diagnosis.

At present, in most cases, accurate prenatal diagnosis requires invasive testing. There is current research into noninvasive prenatal diagnosis using PCR and molecular genetic techniques to examine fetal DNA obtained from maternal blood.

